# Recent Progress in MXene-Based Composites for CO_2_ Conversion: A Mini Review

**DOI:** 10.3390/nano16211400

**Published:** 2026-10-31

**Authors:** Quoc Hai Nguyen, Shalini Sahani, Phan Khanh Thinh Nguyen

**Affiliations:** 1 Group of Applied Research in Advanced Materials for Sustainable Development, Faculty of Applied Sciences, Ton Duc Thang University, Ho Chi Minh City 700000, Vietnam; 2 School of Chemical Engineering, Yeungnam University, Gyeongsan 38541, Gyeongsangbuk-Do, Republic of Korea; shalinisahani@yu.ac.kr; 3 School of Chemical, Biological, and Battery Engineering, Gachon University, 1342 Seongnamdaero, Sujeong-Gu, Seongnam-Si 13120, Gyeonggi-Do, Republic of Korea

**Keywords:** MXene, photocatalytic CO_2_ reduction, two-dimensional materials, photocatalysts

## Abstract

CO_2_ conversion has emerged as a sustainable and environmentally friendly strategy for producing value-added fuels and chemicals while mitigating greenhouse gas emissions. However, the practical implementation of conventional CO_2_ conversion technologies is often hindered by sluggish reaction kinetics, unfavorable thermodynamics, and complex multistep reaction pathways. Since their first discovery in 2011, MXenes have attracted significant attention as promising catalytic materials owing to their unique physicochemical properties, including high electrical conductivity, tunable surface chemistry, large specific surface area, and excellent charge-transfer capability. Consequently, MXene-based photocatalysts have demonstrated remarkable progress in solar-driven CO_2_ conversion applications. This mini-review provides a basic overview of recent advances in MXene-based composites for CO_2_ conversion. First, the fundamental characteristics of MXenes, including their synthesis strategies and key physicochemical properties relevant to photocatalytic CO_2_ conversion, are introduced. Subsequently, recent developments in MXene-based photocatalytic systems for CO_2_ reduction are basically discussed. Finally, the current challenges, future opportunities, and research directions for engineering high-performance MXene-based composites are highlighted. This review aims to provide both newcomers and experienced researchers with a concise understanding of MXene materials and their growing potential in photocatalytic CO_2_ conversion.

## 1. Introduction

MXenes represent an important class of two-dimensional (2D) materials, consisting of transition metal carbides, nitrides, or carbonitrides, and have attracted significant attention from the nanoscience and technology communities since their first synthesis in 2011 [1,2,3]. MXenes generally follow the chemical formula M_n+1_X_n_T_x_ and are derived from their parent MAX phases, where M is an early transition metal, X denotes carbon and/or nitrogen, A is typically an element such as aluminum or silicon, and T_x_ represents surface termination groups (e.g., –OH, –F, –O) [4,5].

Following selective etching of the A layer, the resulting MXenes exhibit a multilayered structure with excellent electrical conductivity and unique physicochemical properties, including mechanical flexibility, intrinsic hydrophilicity, and a large specific surface area. These distinctive characteristics make MXenes promising candidates for a wide range of applications, including biomedicine, energy harvesting and storage, catalysis, and wastewater treatment [6,7,8].

The applications of MXenes are strongly dependent on their synthesis techniques and exfoliation processes, which directly influence their morphology, structure, and physicochemical properties [9,10]. For instance, intercalation involves the insertion of ions or organic/inorganic solvents between each layer of MXene, leading to expanded interlayer spacing and modifications of surface termination groups, thereby altering electrical conductivity and electrochemical behavior in energy harvesting and storage systems.

In electrochemical applications, precise control over the type and distribution of intercalated species is essential to achieve uniform structural and mechanical properties that meet practical performance requirements. In contrast, the exfoliation process focuses on the delamination of multilayered MXenes into single- or few-layer nanosheets. This process significantly increases the accessible surface area and exposes abundant active sites, which are highly beneficial for applications in sensing, catalysis, and adsorption [11,12,13].

The sonication or manual shaking process is commonly employed to weaken interlayer interactions by facilitating the intercalation of guest species between MXene layers. Optimizing exfoliation conditions is crucial for tailoring the structural characteristics and maximizing the performance of Ti_3_C_2_T_x_ in various applications. Numerous studies have systematically investigated exfoliation strategies and the associated parameters, highlighting their strong interdependence and significant impact on the physicochemical properties of MXenes [14].

MXenes have emerged as rising stars among two-dimensional materials owing to their unique metallic-like characteristics compared with other members of the 2D material family [15]. Despite substantial progress, challenges associated with scalable and sustainable synthesis, environmental stability, and the simultaneous optimization of surface reactivity, charge transport, and interfacial interactions remain important barriers to practical implementation. Various MXene compositions, including Cr_2_CT_x_, Ti_3_C_2_T_x_, and V_2_CT_x_, provide broad opportunities for tailoring and optimizing physicochemical properties through surface termination engineering and transition-metal layer modulation. In addition, MXenes exhibit strong light–matter interactions associated with plasmonic behavior, along with high surface affinity toward reactant molecules, making them highly promising candidates for solar-driven CO_2_ conversion applications [16,17,18,19]. Although previous reviews have extensively discussed MXene-based photocatalysts for dye degradation and related energy-conversion processes, further basic analysis is needed to establish explicit relationships between synthesis conditions, structural and morphological characteristics, surface chemistry, and photocatalytic function.

In this work, we summarize the morphological and physicochemical properties of Ti_3_C_2_T_x_, which collectively influence its performance in photocatalytic applications. This review aims to provide a fundamental understanding of MXene preparation strategies and composite integration, highlighting the potential of MXenes as versatile materials for enhancing photocatalytic CO_2_ conversion.

## 2. Synthesis

Ti_3_C_2_T_x_ MXene is commonly derived from the corresponding Ti_3_AlC_2_ MAX-phase precursor via selective chemical etching. Fluoride-based etchants, particularly hydrofluoric acid (HF), are widely used to selectively remove the Al atomic layers. This process is facilitated by the stronger ionic Ti–C bonds relative to the metallic Ti–Al bonds within the MAX-phase structure, leading to the formation of two-dimensional Ti_3_C_2_T_x_ MXene nanosheets. Simultaneously, surface functional groups such as –F, –OH, and =O are generated and anchored onto the MXene surface, producing the characteristic terminated Ti_3_C_2_T_x_ structure that governs its surface chemistry, hydrophilicity, and electronic properties. The chemical reactions occurring during the etching process are summarized as follows:

(1) Selective etching of Al layers: Ti_3_AlC_2_ + 3HF → Ti_3_C_2_ + AlF_3_ + 1.5H_2_
(2) Formation of fluorine terminations: Ti_3_C_2_ + 2HF → Ti_3_C_2_F_2_ + H_2_
(3) Formation of hydroxyl terminations: Ti_3_C_2_ + 2H_2_O → Ti_3_C_2_ (O/OH)_2_ + H_2_

Intercalation and exfoliation of Ti_3_C_2_T_x_ nanosheets involve the insertion of ions, compounds, or guest molecules into the interlayer spaces of MXene without disrupting its structural integrity. This strategy plays a critical role in tailoring interlayer electrostatic interactions, as positively charged intercalants interact with the negatively charged MXene surfaces, thereby enhancing ion transport and improving electrical conductivity. The intrinsic hydrophilicity of MXenes is further enhanced through the combined effects of surface termination groups and intercalated species. The insertion of cationic species, such as Mg^2+^, Na^+^, and K^+^, into the interlayer spaces can be facilitated under an applied potential, which is highly beneficial for energy harvesting and storage applications. By weakening van der Waals interactions between adjacent layers, the exfoliation process serves as an effective strategy to expand interlayer spacing, thereby enhancing ion transport, charge-carrier mobility, and water-interaction behavior.

Surface functional groups play a key role in determining the electronic properties, surface reactivity, and overall performance of MXenes. Tailoring these functional groups and their local atomic environments is essential because surface terminations can be modified through interactions with molecules or guest ions. Such modifications can tune the surface charge distribution and work function of MXenes, thereby influencing interfacial charge-transfer dynamics and ion accessibility.

Exfoliation increases the exposure of active surface atoms and enhances the accessibility of surface titanium sites, thereby facilitating post-synthetic modification. In particular, the concentration of –F surface groups introduced during synthesis significantly influences hydrogen-bonding interactions and ion-exchange behavior in electrolytes.

**Figure 1:**
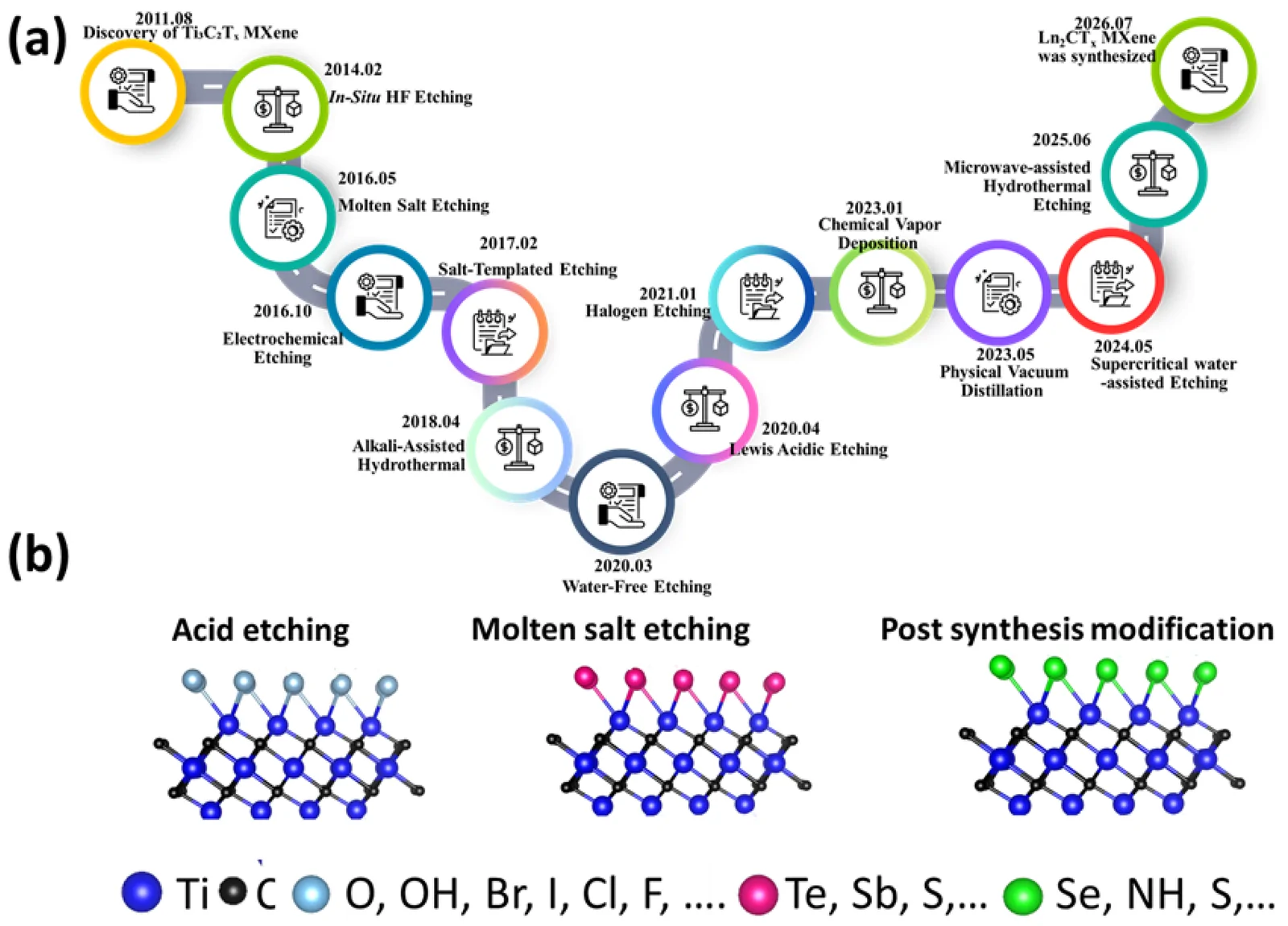
(**a**) Timeline of Ti_3_C_2_T_x_ MXene synthesis technologies from 2011 to 2026, illustrating the evolution from conventional chemical etching to advanced approaches, including electrochemical etching, molten-salt etching, hydrothermal etching, vapor-assisted etching, microwave-assisted hydrothermal etching, and supercritical water-assisted etching. (**b**) Schematic illustration of the surface functional groups associated with different etching methods and post-synthesis modification. Reproduced from Ref. [3] under the terms of the Creative Commons Attribution (CC BY) license.

As shown in Figure 1, the synthesis of Ti_3_C_2_T_x_ MXene has undergone remarkable development since its initial discovery in 2011. A wide range of synthesis strategies have been reported, including chemical, electrochemical, hydrothermal, vapor-assisted, and rapid-heating methods, reflecting continuous efforts to improve synthesis efficiency and material quality. Although each technique exhibits unique strengths and inherent limitations, challenges related to scalability, production cost, environmental sustainability, and process safety remain critical. For instance, the conventional HF etching method typically produces MXenes with mixed surface terminations, which may limit electronic tunability and complicate further functional modification because of the Ti–F bonding. In contrast, Lewis acidic molten-salt strategies enable the synthesis of MXenes with more uniform halide terminations that are weaklier bound, thereby providing greater flexibility for surface engineering and property optimization (Table 1). Consequently, future industrialization of MXene production will require systematic optimization together with comprehensive techno-economic and environmental assessments.

The choice of MXene synthesis method determines not only the etching efficiency and resulting morphology but also the surface termination chemistry, which can substantially influence photocatalytic CO_2_ reduction. In particular, the surface –F, –OH, and –O groups introduced during etching and subsequent processing modify the local electronic structure, surface polarity, work function, and interfacial charge-transfer characteristics of MXenes. These properties collectively affect CO_2_ adsorption, the stabilization of reaction intermediates, and the utilization of photogenerated charge carriers.

HF-based etching commonly produces MXenes bearing mixed –F, –OH, and –O terminations, with their relative proportions depending on the etching and post-treatment conditions. These terminations can influence catalytic behavior through different mechanisms. Oxygen-containing terminations may modify surface electron density and provide favorable interfacial environments for CO_2_ adsorption and activation. Hydroxyl groups can contribute to hydrogen bonding and proton-coupled surface reactions, whereas fluorine terminations can alter the local electrostatic environment and electronic structure. However, the presence of any individual termination does not necessarily guarantee enhanced CO_2_ reduction, because excessive surface coverage may block adsorption sites or alter the energetics of key intermediates. Accordingly, the catalytic effects of HF-derived terminations should be evaluated in terms of their composition, coverage, and interaction with the coupled photocatalyst.

In contrast, molten-salt etching provides an alternative route for tailoring MXene surface chemistry through the selection of salt composition and reaction conditions. Depending on the salt and processing environment, this approach can produce surface terminations that differ from those obtained through conventional HF-based etching, potentially enabling more deliberate control of the interfacial electronic structure. Nevertheless, molten-salt etching should not be assumed to produce chemically uniform or exclusively –F-free surfaces in every case; the resulting termination distribution depends on the specific synthesis chemistry. For photocatalytic CO_2_ reduction, the relevant advantage is therefore the possibility of tuning the surface composition to optimize CO_2_ adsorption, charge redistribution, and the stabilization of intermediates involved in pathways toward CO, HCOOH, CH3OH, or CH4.

Ultimately, the relationship between synthesis procedure and catalytic performance is mediated by the surface structure and electronic properties of the resulting MXene. Surface terminations can alter the work function and interfacial band alignment of MXene–semiconductor composites, thereby influencing electron transfer and charge-carrier recombination. Simultaneously, the chemical nature of the surface determines the adsorption configuration and binding strength of CO_2_-derived intermediates, which can affect reaction pathways and product selectivity. Therefore, optimizing MXenes for photocatalytic CO_2_ reduction requires coordinated control of termination chemistry, defect structure, and interfacial coupling, rather than simply maximizing etching efficiency, exfoliation yield, or production scale.

## 3. Properties

MXenes exhibit composition- and surface-termination-dependent electronic properties. Ti_3_C_2_T_x_ generally exhibits metallic behavior, with its electronic structure influenced by the hybridization of transition-metal d orbitals with C 2p and, where applicable, surface-termination-derived O 2p orbitals. Although many MXenes exhibit metallic characteristics, the electronic structures of other MXene compositions may differ, including semiconducting behavior in specific systems. Therefore, their band structures and band gaps should be discussed in the context of the specific MXene composition and surface chemistry. Among various surface terminations, Ti_3_C_2_O_x_ exhibits a higher density of states near the Fermi level compared with –OH- and –F-terminated Ti_3_C_2_T_x_, resulting in enhanced electrical conductivity. Based on density functional theory (DFT) calculations, the bandgap energies of MXenes have been estimated to range from approximately 0.05 to 2 eV, as summarized in Table 2 [20,21,22].

One of the key properties of Ti_3_C_2_T_x_ MXenes that strongly influences their electronic band structure and density of states is their optical behavior. Ti_3_C_2_T_x_ exhibits effective light absorption arising from interband electronic transitions in the ultraviolet region. In addition, MXenes contain abundant free electrons and demonstrate plasmonic behavior associated with localized surface plasmon resonance (LSPR), which contributes to enhanced light transmission and reflection in the visible and infrared regions. Thinner MXene nanosheets may exhibit enhanced SPR responses, potentially facilitating light harvesting and charge-carrier transfer. These properties make ultrathin MXene a promising photocatalytic cocatalyst for improving photocatalytic performance [23].

**Figure 2:**
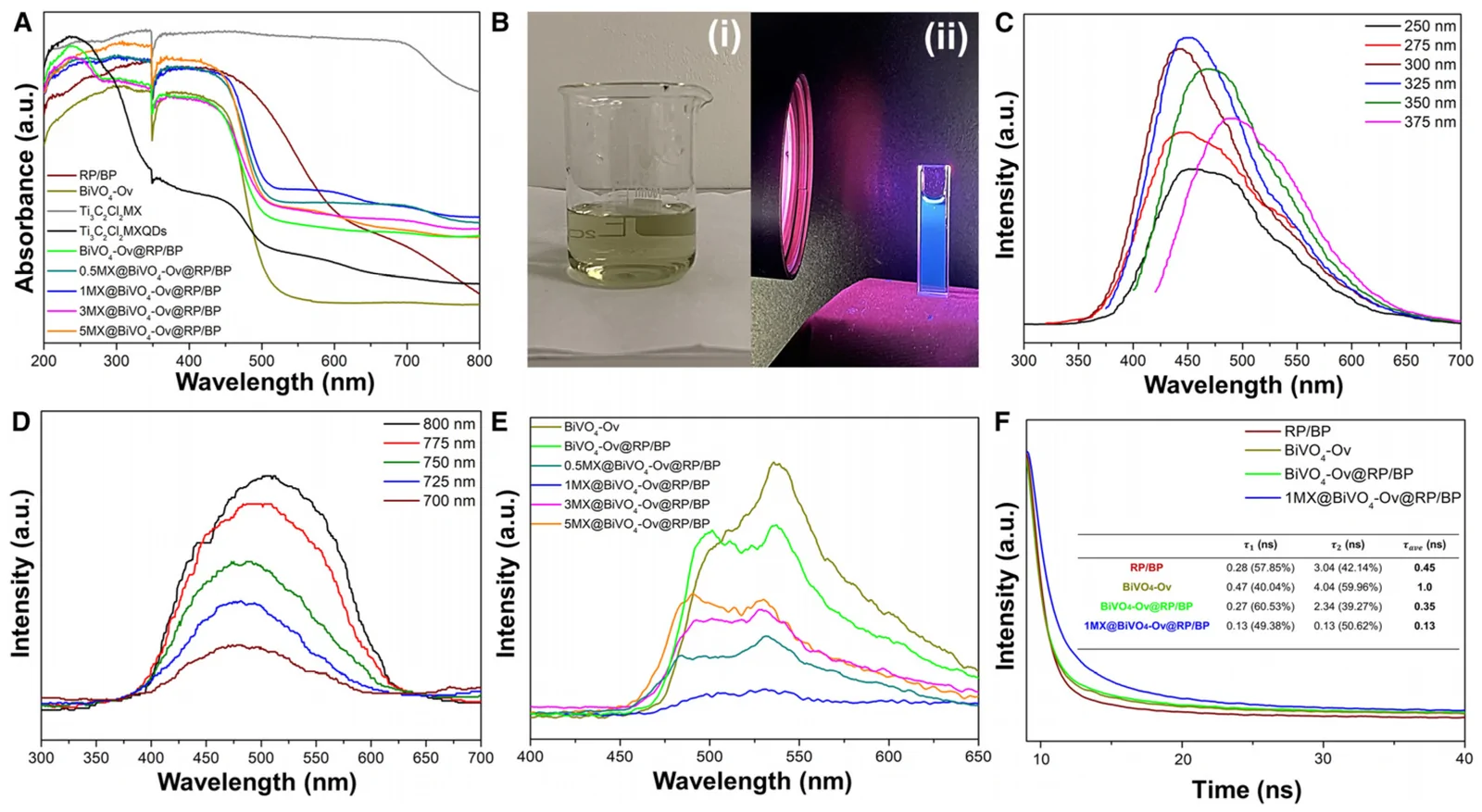
Optical and photoluminescence characterization of MXene-based photocatalysts: (**A**) UV–vis absorption spectra; (**B**) photographs under ambient and UV-light irradiation; (**C**) excitation-dependent PL spectra; (**D**) upconversion PL spectra; (**E**) steady-state PL spectra; and (**F**) TRPL decay curves with corresponding average lifetimes. Reproduced from Ref. [29] under the terms of the Creative Commons Attribution (CC BY) license.

Notably, MXenes exhibit superior direct-current and optical conductivity compared with conventional carbon-based nanomaterials such as carbon nanotubes and graphene, owing to their low sheet resistance (~201 Ω sq^−1^) and high optical transmittance, reaching approximately 93% for ultrathin films (~4 nm) and 29% for thicker films (~88 nm) [24]. Furthermore, the optical and plasmonic properties of MXenes can be effectively tailored by modifying the synthesis conditions and varying the transition metal composition of the parent MAX phase. Such tunable optical characteristics are highly advantageous for enhancing the efficiency of photocatalytic CO_2_ reduction systems [25,26,27]. Moreover, Ti_3_C_2_T_x_ quantum dots (QDs) exhibit photoluminescence properties owing to quantum confinement and edge effects [28]. As displayed in Figure 2, the ultrasmall Ti_3_C_2_T_x_ QDs produce strong luminescence under optical excitation, highlighting their unique optical characteristics [29]. MQDs and MXene NSs exhibit complementary characteristics in photocatalytic CO_2_ reduction: MQDs offer abundant exposed edge/defect sites and short charge-transfer pathways, whereas NSs provide extended conductive networks and large interfacial contact areas. These complementary properties make dimensional engineering of MXene a promising strategy for tailoring CO_2_ adsorption, charge separation, and interfacial reaction kinetics.

Near-free-electron (NFE) states also play an important role in determining electron mobility and transport behavior in Ti_3_C_2_T_x_ MXenes. Ti- and Hf-derived –OH terminated MXenes exhibit NFE states close to the Fermi level, resulting in higher charge-carrier mobility compared with their O-terminated counterparts. These features demonstrate that the electrical conductivity of MXenes can be effectively tuned through surface termination engineering, with reported conductivities reaching approximately 10^4^ S cm^−1^ and carrier mobilities on the order of 1 cm^2^V^−1^s^−1^.

Surface termination groups also strongly influence the optical properties of MXenes by modulating NFE states. For example, –OH-terminated MXenes exhibit NFE states close to the Fermi level, which can enhance optical transparency and facilitate electron transport with reduced scattering compared with –F- or –O-terminated counterparts. In contrast, =O-terminated MXenes generally show stronger light absorption and can promote electronic transitions more effectively than other surface terminations.

The magnetic properties of MXenes can be analyzed through their magnetic moments, which may exhibit ferromagnetic or non-magnetic behavior depending on the surface terminations and composition. MXenes terminated with =O groups generally demonstrate stronger ferromagnetic characteristics than those terminated with –OH or –F groups. This behavior is associated with differences in electronegativity among the functional groups (–OH < =O < –F), which influence the nature of chemical bonding and electronic interactions. In particular, =O terminations tend to form more anisotropic covalent bonds, facilitating stronger overlap between the O 2p orbitals and transition-metal d orbitals, whereas –OH- and –F-terminated MXenes are more likely to exhibit isotropic bonding characteristics.

Furthermore, increasing electronegativity of the surface functional groups can enhance electron localization effects, thereby influencing the magnetic properties of MXenes. Most of these magnetic behaviors have primarily been predicted through DFT calculations, and further experimental investigations are still required to validate the proposed spin–orbit coupling mechanisms and anisotropic/isotropic bonding effects.

As summarized in Table 3, MQDs and MXene NSs exhibit distinct structural and physicochemical characteristics that influence their suitability for photocatalytic CO_2_ reduction. However, these differences should be interpreted in terms of their functional roles within the complete photocatalytic architecture rather than as intrinsic evidence of the superiority of one morphology over the other. Accordingly, MQDs are particularly promising when localized interfacial interactions, short transport distances, and accessible edge-associated sites are desirable, whereas NSs are advantageous when extended conductive pathways and large interfacial contact areas are required. These complementary characteristics suggest different design strategies for CO_2_ photoreduction. For instance, MQDs can be dispersed or anchored onto semiconductor surfaces to facilitate interfacial charge transfer, while NSs can serve as conductive cocatalysts or form two-dimensional heterojunctions that promote charge transport. Nevertheless, the effectiveness of either architecture depends on surface chemistry, interfacial energy alignment, and structural accessibility. In particular, MQD aggregation and NS restacking can diminish the expected benefits, highlighting the importance of controlling dispersion and constructing accessible hierarchical interfaces.

## 4. Applications of MXene-Based Materials

### 4.1. Photocatalytic CO_2_ Reduction

**Figure 3:**
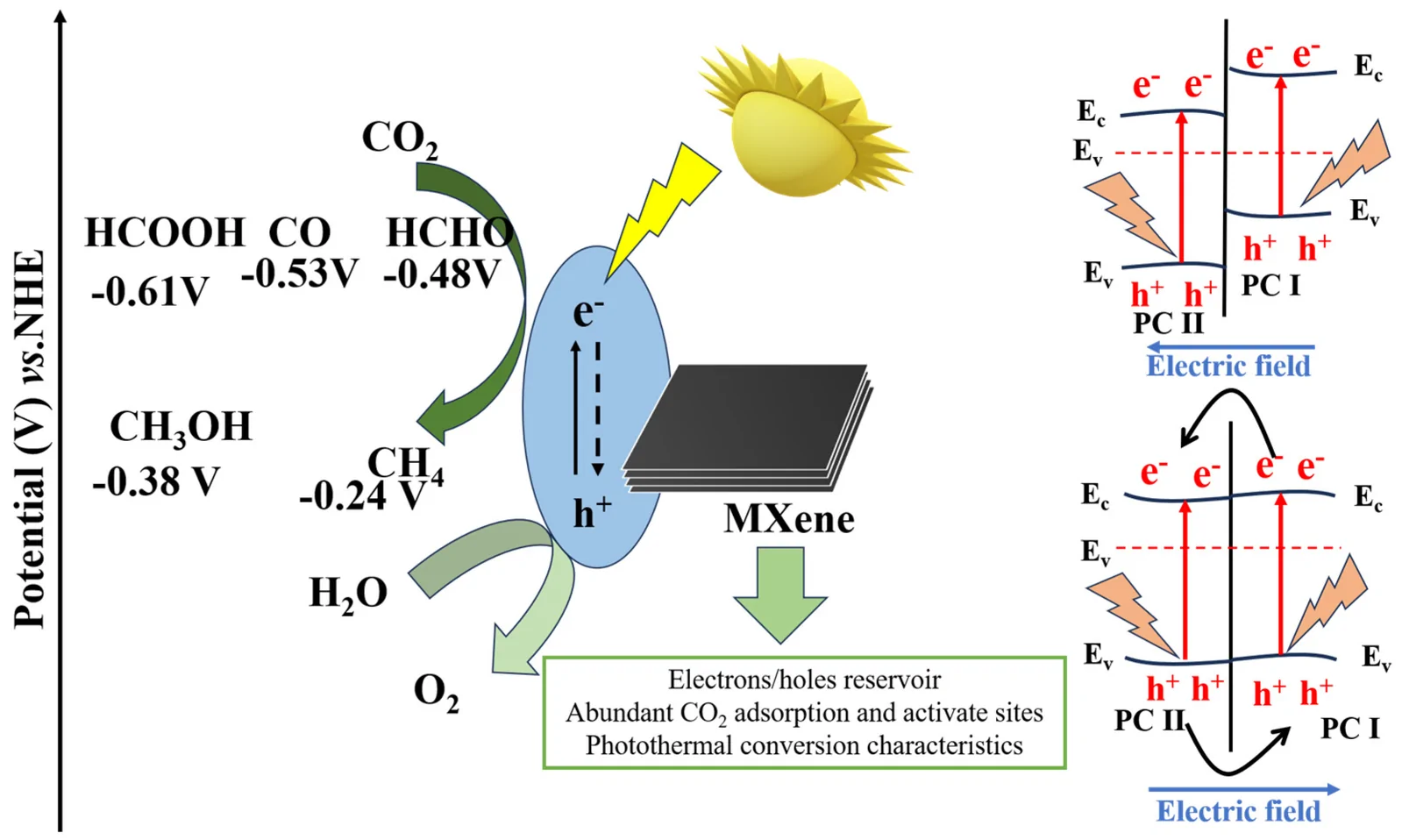
Schematic illustration of the proposed mechanism of MXene-based photocatalysts for photocatalytic CO_2_ reduction, highlighting photogenerated charge separation, interfacial electron transfer, CO_2_ adsorption and activation, and the synergistic electron-reservoir and photothermal effects of MXene.

In photocatalytic systems, MXene can serve as an efficient electron reservoir and conductive charge-transfer medium, facilitating the separation and migration of photogenerated charge carriers and suppressing electron–hole recombination [30]. These properties are closely associated with the high electrical conductivity, tunable surface chemistry, and favorable work function of MXenes. As illustrated in Figure 3, MXenes can enhance photocatalytic CO_2_ reduction through four distinct but potentially complementary mechanisms:

(i) MXenes as cocatalysts for trapping and transporting photogenerated electrons.

Under light irradiation, the semiconductor absorbs photons and generates electron–hole pairs. When the semiconductor and MXene form an appropriate interface, differences in their work functions and interfacial energy levels can drive electron redistribution and establish an interfacial electric field. In suitable systems, the resulting Schottky barrier facilitates electron transfer from the semiconductor to the MXene and suppresses charge-carrier recombination. Owing to their high electrical conductivity, MXenes can act as electron-accepting cocatalysts and charge-transfer pathways, thereby increasing the availability of electrons for surface CO_2_ reduction. However, the direction and efficiency of charge transfer depend on the specific interfacial energetics, surface terminations, and heterostructure configuration; thus, the formation of a Schottky junction or a particular charge-transfer pathway should be established for each system rather than assumed universally.

(ii) LSPR-induced hot-electron generation and injection.

Certain MXenes exhibit localized surface plasmon resonance, which can generate energetic charge carriers under suitable optical excitation. Following plasmon excitation, non-radiative relaxation can produce non-equilibrium hot electrons that may be injected into adjacent semiconductor or catalytic phases when the interfacial energy alignment and transfer kinetics are favorable. These electrons can provide an additional pathway for populating reactive electronic states and promoting CO_2_ reduction. Unlike conventional cocatalytic electron transfer, which primarily involves the redistribution and transport of photogenerated carriers, LSPR-mediated hot-electron injection originates from plasmon excitation and subsequent carrier relaxation. Its contribution should therefore be distinguished from ordinary charge separation and verified through appropriate optical and time-resolved spectroscopic measurements.

(iii) Interfacial-interaction-induced defects and enhanced CO_2_ activation.

Strong interactions between MXenes and supported semiconductor phases can modify local atomic structures, interfacial charge distributions, and defect formation energies. For example, MXene-supported CuInS_2_ nanosheet arrays have been reported to exhibit sulfur vacancies associated with a strong support effect and interfacial charge transfer. These vacancies facilitate CO_2_-to-CO photoreduction by lowering the calculated energy barrier for a key intermediate-conversion step. Such defect sites can alter CO_2_ adsorption, electronic-state distributions, and the stabilization of reaction intermediates, thereby influencing reaction kinetics and product selectivity. Nevertheless, the effect of defects is composition- and concentration-dependent: an appropriate defect population may promote catalytic activity, whereas excessive defects can introduce recombination centers or destabilize the catalyst.

(iv) Synergistic photothermal enhancement.

MXenes can also contribute to light-to-heat conversion, increasing the local temperature of a photocatalytic system under suitable irradiation conditions. The resulting thermal contribution may accelerate temperature-dependent surface reactions, facilitate intermediate conversion, and improve the utilization of photogenerated carriers. In plasmonic systems, optical absorption can contribute to both hot-carrier generation and photothermal heating; however, these are distinct processes and should not be treated as interchangeable explanations for enhanced activity. The photothermal contribution should be evaluated by comparing catalytic performance under controlled temperature conditions and, where feasible, separating thermal effects from non-thermal photoinduced processes.

These four mechanisms can operate simultaneously in an appropriately designed MXene-based photocatalyst, but their relative contributions depend on MXene composition, surface terminations, interfacial structure, optical properties, and reaction conditions. Accordingly, improved photocatalytic CO_2_ reduction should be attributed to experimentally supported mechanisms rather than inferred solely from increased activity [31,32].

Although MXenes are non-noble-metal materials, some MXenes have been reported to exhibit plasmonic characteristics. Their localized surface plasmon resonance can contribute to photocatalytic enhancement through hot-electron generation and injection, local electromagnetic-field enhancement, and photothermal effects [33]. In addition, the two-dimensional lamellar structure of MXenes provides a high surface area and abundant interfacial sites for the dispersion and growth of semiconductor nanostructures. The resulting MXene/semiconductor heterostructures can facilitate interfacial charge transfer while reducing particle aggregation and providing accessible active sites. These structural features are beneficial for reactant adsorption, mass transfer, and subsequent CO_2_ conversion. The concept of strong metal–support interaction traditionally refers to the strong interaction between metallic active phases and reducible oxide supports, which can modify the electronic structure, surface properties, and catalytic behavior of the active metal. In MXene-based photocatalytic systems, strong interfacial interactions between MXenes and semiconductor components can similarly induce substantial electronic redistribution and modify the surface defect structure, thereby influencing CO_2_ adsorption and activation.

For example, the formation of sulfur vacancies has been reported in CuInS_2_/Ti_3_C_2_T_x_ heterostructures, where the relatively work function of MXene promotes interfacial electron transfer from CuInS_2_ to Ti_3_C_2_T_x_ MXene [34]. This electronic interaction can facilitate sulfur-vacancy formation during the formation of CuInS_2_ and induce charge redistribution at the CuInS_2_/Ti_3_C_2_T_x_ interface. The resulting electronic modulation can alter the surface binding characteristics and adsorption behavior toward CO_2_, thereby facilitating CO_2_ activation and subsequent photoreduction. It is well recognized that strong interactions between semiconductor supports and metallic active components can significantly enhance catalytic performance and modulate the electronic structure of composite systems. In particular, electron paramagnetic resonance (EPR) analysis confirmed that the incorporation of MXene promoted the formation of sulfur vacancies in CuInS_2_/MXene photocatalysts. DFT calculations further supported the experimental observations, revealing that MXenes possess a high work function and strong electron-withdrawing capability, which facilitate the generation of sulfur vacancies [34]. Moreover, various metal sulfides containing sulfur vacancies have been successfully integrated into MXene substrates, resulting in enhanced photocatalytic CO_2_ reduction performance. In addition, X-ray photoelectron spectroscopy (XPS) analyses demonstrated significant electron redistribution between the catalyst surface and adsorbed CO_2_ molecules, which is beneficial for CO_2_ adsorption and activation.

**Figure 4:**
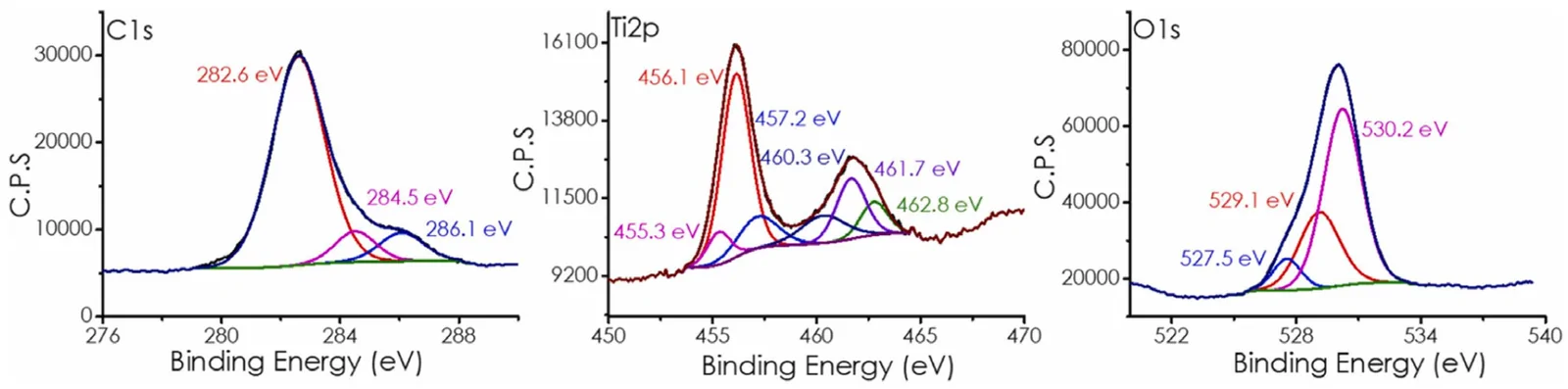
High-resolution XPS spectra of Ti_3_C_2_T_x_ MXene: including C 1s, Ti 2p, and O 1s, showing the characteristic bonding states and surface functional groups. Reproduced from Ref. [35] under the terms of the Creative Commons Attribution (CC BY) licence.

XPS was employed to investigate the surface chemical composition and chemical states of MXene, as shown in Figure 4. The high-resolution C 1s spectrum exhibited three major components, which were assigned to Ti–C bonds (approximately 283 eV), sp^2^-hybridized C–C bonds (approximately 284–285 eV), and C–O bonds (approximately 286 eV). Furthermore, the O 1s spectrum revealed oxygen-containing surface species, including Ti–OH groups, indicating the presence of hydroxyl terminations on the MXene surface. The high-resolution Ti 2p spectrum exhibited characteristic spin–orbit-split doublets, providing information on the chemical states of Ti, including Ti–C and oxidized Ti species [35]. These surface chemical features may influence CO_2_ adsorption and interfacial charge transfer, potentially contributing to the photocatalytic reduction of CO_2_.

He et al. [36] synthesized a TiO_2_/g-C_3_N_4_@MXene composite via electrostatic self-assembly for photocatalytic CO_2_ reduction. Time-resolved photoluminescence (TRPL) measurements revealed a systematic decrease in the average photoluminescence lifetime of the TiO_2_/g-C_3_N_4_ composite (T-CN) compared with that of pristine g-C_3_N_4_ (CN), suggesting accelerated charge-carrier dynamics following heterojunction formation. Furthermore, steady-state photoluminescence (PL) spectra showed a reduced emission intensity in the composite, indicating suppressed radiative electron–hole recombination and suggesting improved charge-carrier separation. The incorporation of MXene, which possesses high electrical conductivity and electronic states near the Fermi level, can facilitate interfacial charge transfer and promote the extraction of photogenerated electrons. In particular, oxygen-containing surface terminations (–O/–OH) may contribute to interfacial interactions and modulate the electronic properties of MXene, thereby facilitating charge-carrier separation and enhancing photocatalytic CO_2_ reduction [37]. Electrochemical impedance spectroscopy (EIS) further supported the enhanced interfacial charge-transfer kinetics, as evidenced by the smaller semicircle diameter in the Nyquist plot of Ru@Ti_3_CN@TiO_2_ compared with that of Ti_3_CN@TiO_2_, indicating a lower charge-transfer resistance [38]. Furthermore, Ru@Ti_3_CN@TiO_2_ exhibited a substantially higher photocurrent density, suggesting more efficient separation and transport of photogenerated charge carriers under light irradiation. Based on these results, the proposed mechanism highlights the important role of MXene as an electron acceptor and charge-transfer mediator, facilitating electron extraction and suppressing electron–hole recombination. The incorporation of Ru further promotes interfacial charge transfer, thereby enhancing the overall photocatalytic performance.

As the first experimentally reported MXene, Ti_3_C_2_T_x_ has been extensively investigated as a component of composite photocatalysts for CO_2_ photoreduction, as summarized in Table 4. Various value-added carbon products, including CO, CH_4_, HCOOH, and CH_3_COOH, have been reported. The product distribution and selectivity depend on several factors, including the synthesis methods, physicochemical properties, structural configurations, and metal compositions of the Ti_3_C_2_T_x_-based photocatalysts, as well as the reaction conditions.

**Figure 5:**
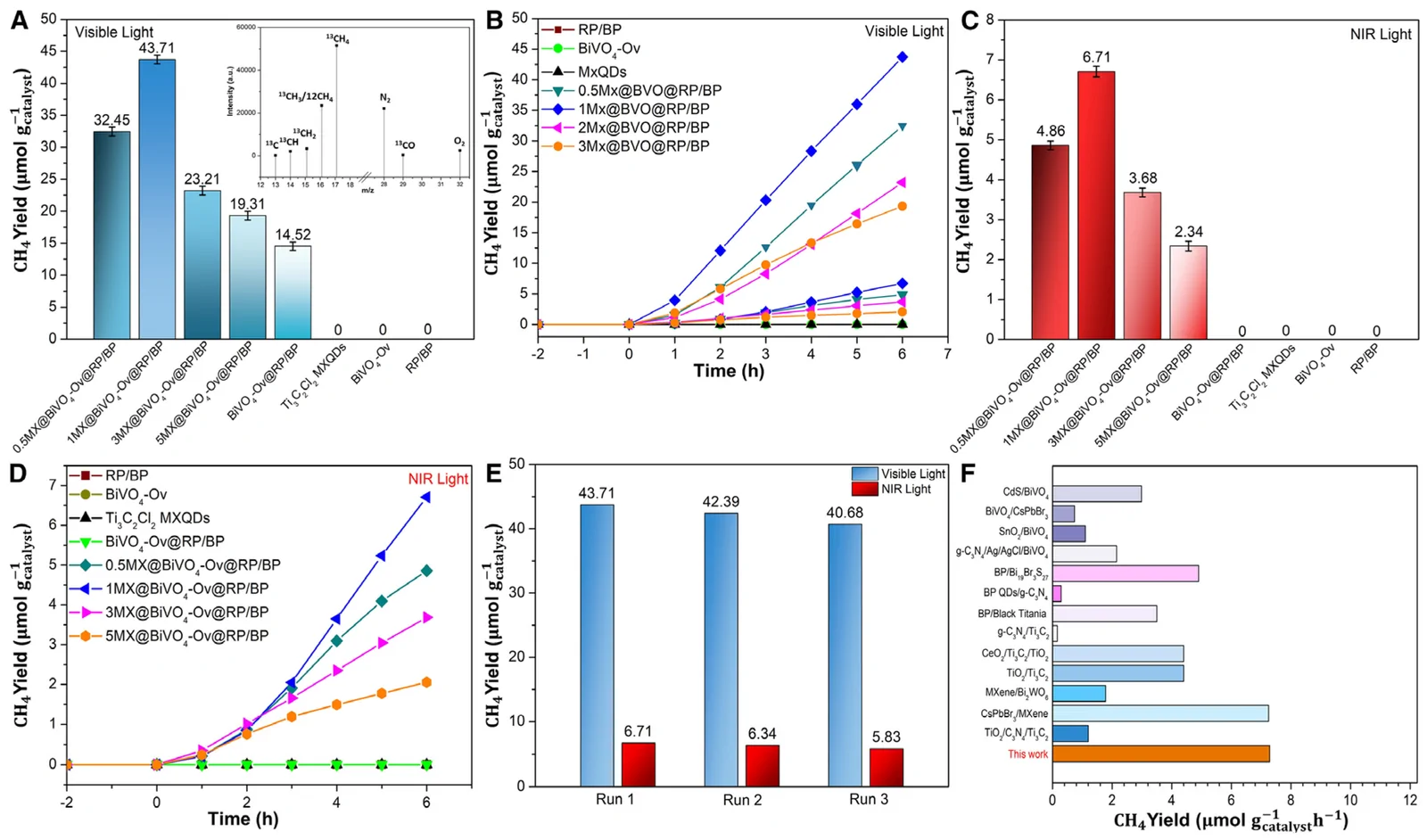
Photocatalytic CO_2_ reduction performance of Ti_3_C_2_T_x_@BiVO_4_–Oᵥ@RP/BP under visible and NIR light irradiation: (**A**–**D**) CH_4_ evolution, (**E**) comparison of CH_4_ yields, and (**F**) comparison with previously reported photocatalysts. Reproduced from Ref. [29] under the terms of the Creative Commons Attribution (CC BY) license.

Yang et al. synthesized g-C_3_N_4_/Ti_3_C_2_T_x_ composites for photocatalytic CO_2_ conversion, achieving CH_4_ and CO production yields of approximately 0.044 μmol g^−1^h^−1^ and 5.19 μmolg^−1^h^−1^, respectively [51]. By directly annealing urea with MXene, an intimate 2D/2D heterojunction with a sandwich-like structure was successfully constructed, which facilitated efficient photogenerated charge separation and suppressed electron–hole recombination. Owing to the prolonged lifetime of charge carriers and enhanced CO_2_ activation, the CO_2_ conversion performance of the composite was enhanced by nearly eight times compared with pristine g-C_3_N_4_. As shown in Figure 5, the integration of MXene with BiVO_4_–Oᵥ@RP/BP facilitates the generation and migration of photogenerated charge carriers, thereby enhancing the photocatalytic CO_2_ reduction performance. Accordingly, CH_4_ yields of 43.71 and 6.71 μmol g^−1^ were achieved after 6 h of irradiation under visible light (>400 nm) and NIR light (>700 nm), respectively [29]. Similarly, other 2D/2D heterostructures, such as g-C_3_N_4_/Ti_3_C_2_T_x_ composites and porous g-C_3_N_4_/V_2_C systems, have also demonstrated excellent photocatalytic CO_2_ reduction performance [52].

MXene and its combinations with zero-, one-, and two-dimensional catalysts provide maximized interfacial contact and strong interactions between the active components, thereby facilitating charge transfer and enhancing photocatalytic activity. In addition, controlling the ratio of surface termination groups (–F, –O, and –OH) can effectively tailor the density of active sites, which strongly influences the hydrogen production yield of photocatalysts. Among various nanostructures, zero-dimensional (0D) quantum dots generally exhibit superior photocatalytic performance due to their unique physicochemical properties, including large specific surface area, tunable optoelectronic characteristics, and enhanced catalytic behavior. Zeng et al. demonstrated that Cu_2_O/MXene quantum dots exhibited significantly higher CO_2_ conversion efficiency than hierarchical Cu_2_O nanowire-based structures, owing to improved charge separation and increased active surface sites [53].

The SEM results revealed that the morphology of Cu_2_O nanowires was well preserved after the incorporation of MXene quantum dots. This structural stability can be attributed to the electrostatic self-assembly process, which involved the adsorption of negatively charged poly(sodium 4-styrenesulfonate) onto the Cu_2_O nanowire surface, followed by the grafting of positively charged polyethyleneimine onto the MXene quantum dots. The resulting electrostatic interactions facilitated the uniform integration of MXene quantum dots onto the Cu_2_O framework. As an optimized photocatalyst, the MXene QDs/Cu_2_O NWs/Cu composite exhibited outstanding photocatalytic performance for CO_2_ reduction, achieving a CH_3_OH yield of approximately 154 ppm after six consecutive reaction cycles while retaining 89% of its initial activity. This yield was nearly eight-fold higher than that of the pristine Cu/Cu_2_O catalyst and approximately two-fold greater than that of the MXene nanosheet/Cu_2_O NWs/Cu counterpart.

DFT calculations further demonstrated that MXene quantum dots possess a high density of electronic states in the vicinity of the Fermi level, which enhances electrical conductivity and facilitates rapid electron transport. Moreover, electrochemical impedance spectroscopy (EIS) analysis revealed that the MXene QDs/Cu_2_O NWs/Cu photocatalyst exhibited the lowest charge-transfer resistance among all investigated samples. The significantly reduced Nyquist semicircle diameter confirms enhanced charge mobility and more efficient interfacial electron-transfer kinetics compared with those of the individual components and reference catalysts.

**Figure 6:**
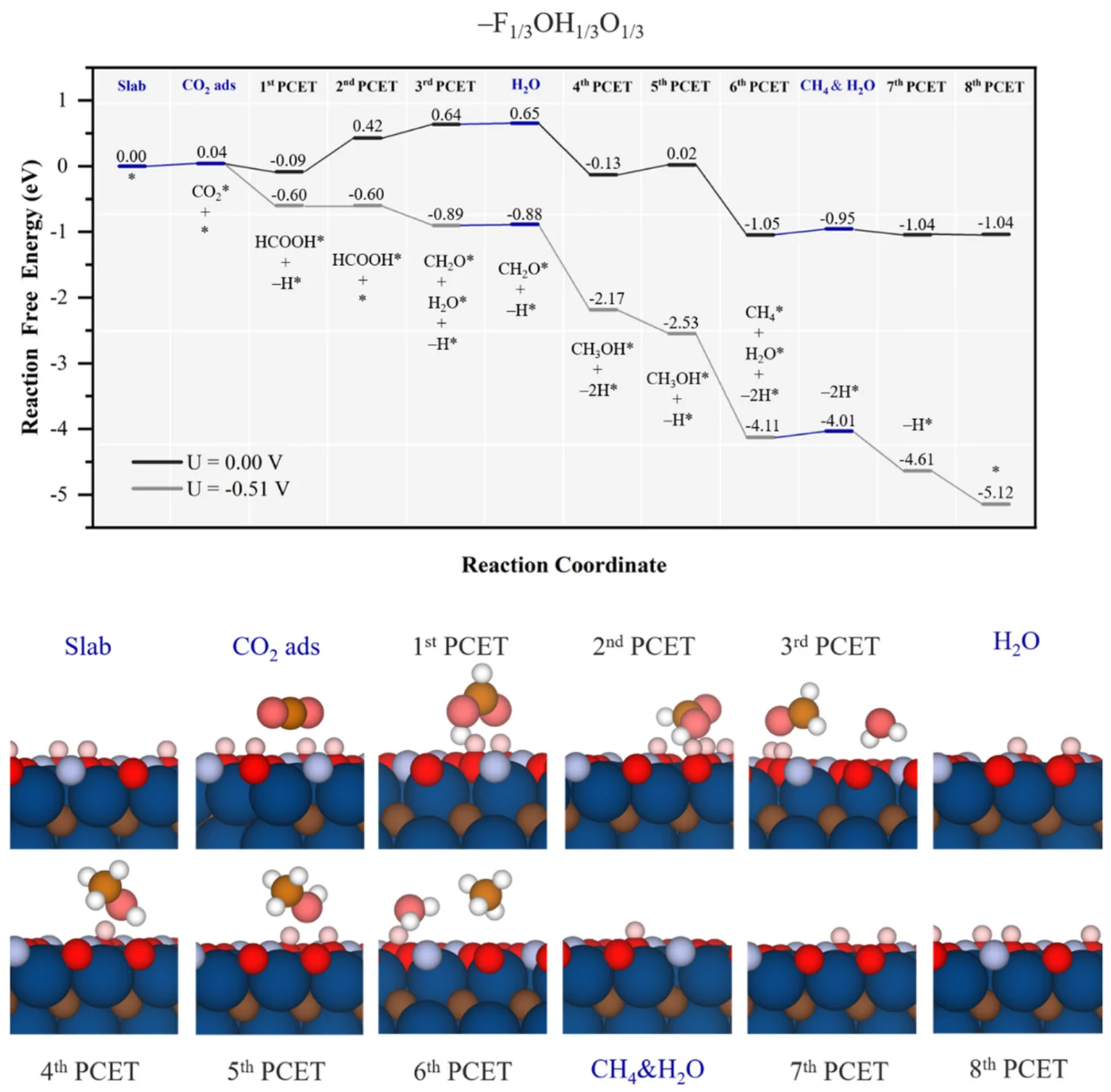
Proposed reaction pathway for photocatalytic CO_2_ reduction to CH_4_ via successive proton-coupled electron-transfer (PCET) steps, showing the calculated reaction free-energy profiles at applied potentials of U = 0.00 V and U = −0.85 V. The corresponding structural models illustrate the sequential transformation of adsorbed CO_2_ through reaction intermediates toward CH_4_ and H_2_O. The asterisk (*) denotes an adsorption site on the catalyst surface, while species marked with an asterisk (e.g., CO_2_, HCOOH, and CH_3_OH) represent surface-adsorbed intermediates. Reproduced from Ref. [55] under the terms of the Creative Commons Attribution (CC BY) licence.

Although significant progress has been achieved in photocatalytic CO_2_ reduction a deeper understanding of the intrinsic mechanisms governing CO_2_ adsorption, activation, and conversion remains essential for the rational design of MXene-based composites. Theoretical calculations have predicted favorable CO_2_ adsorption or dissociation on multiple MXene surfaces [54]. highlighting their potential for solar-driven CO_2_ conversion. However, these predictions often rely on idealized surface models and may not fully reflect the mixed surface terminations, defects, and structural heterogeneity of experimentally synthesized MXenes. Therefore, the predicted adsorption behavior and reaction pathways must be critically evaluated against experimental evidence to establish reliable structure–activity relationships. As shown in Figure 6, the negative Gibbs free energy associated with CO adsorption on MXene surfaces facilitates the thermodynamically favorable hydrogenation of CO* intermediates, thereby promoting subsequent CO_2_ reduction reactions [55]. In fact, thermodynamic favorability does not guarantee experimentally observable catalytic activity or product selectivity. Differences may arise from surface terminations, defects, reaction kinetics, and operating conditions. Therefore, the predicted reaction pathways, product selectivity, and relative catalytic performance remain theoretical predictions awaiting experimental verification.

### 4.2. Other Applications

Ti_3_C_2_T_x_ MXenes have been investigated in various environmental applications because of their large specific surface area, tunable surface terminations, and distinctive interfacial properties. For example, their surface functional groups can influence water adsorption, ion transport, and interactions with dissolved contaminants. These findings highlight the importance of surface chemistry in determining interfacial interactions and molecular adsorption. In the context of photocatalytic CO_2_ conversion, similar principles are relevant to the adsorption and activation of CO_2_ and H_2_O, the binding of reaction intermediates, and interfacial charge transfer. Therefore, understanding and controlling the surface terminations of Ti_3_C_2_T_x_, including –O, –OH, and –F, may provide useful strategies for tailoring its interaction with reactants and optimizing the performance of MXene-based photocatalysts [20].

In addition, the photocatalytic performance of various photocatalysts has been systematically investigated. Engineered MXene-based composites exhibit significantly enhanced photocatalytic activity compared with pristine MXene and their corresponding counterpart materials. For instance, ZnO coupled with an MXene underlayer (ZnO/MXene) demonstrated an improved photocurrent density of 1.40 mA cm^−2^ at 1.22 V vs. RHE, which is substantially higher than that of pristine MXene (0.88 mA cm^−2^). The enhanced photocatalytic efficiency can be attributed to the role of MXene as an effective conductive support and interfacial platform for ZnO growth, which facilitates charge separation and accelerates interfacial electron transport. In addition, the incorporation of MXene promotes favorable structural transformation and increases the number of active reaction sites [56].

The photoelectrochemical (PEC) performance of MXene-based photocatalysts has been evaluated through hydrogen evolution, CO_2_ photoreduction, and pollutant degradation tests, demonstrating their promising potential for sustainable energy conversion and environmental remediation applications. In addition, Ti_3_C_2_T_x_-based electrocatalysts have attracted considerable attention as promising candidates for water-splitting applications. In general, water splitting involves two half-reactions, namely the hydrogen evolution reaction (HER) and the oxygen evolution reaction (OER), which are responsible for hydrogen and oxygen production, respectively.

For example, Co-CoO@MXene electrocatalysts exhibited a low HER overpotential of 113 mV at a current density of 10 mA cm^−2^, together with a small Tafel slope of 57 mV dec^−1^, indicating favorable electrocatalytic kinetics [57]. Similarly, single atomic Ru@MXene composites demonstrated an OER overpotential of 290 mV with a Tafel slope of 37.9 mV dec^−1^, significantly outperforming pristine MXene and conventional metal-based catalysts owing to their accelerated charge-transfer kinetics and enhanced catalytic activity [58].

In another work, Cu@ZnO/MXene composites were synthesized via a facile preparation strategy for CO_2_ electrocatalytic reduction applications. The strong interfacial interaction between ZnO and Cu@MXene enhances electronic coupling within the composite, facilitating electron transfer from Ti_3_C_2_T_x_ to the Cu active sites. This interfacial charge redistribution may contribute to the improved electrocatalytic performance [59]. Moreover, the relatively low work function (5.08 eV) facilitated the formation of the *COOH intermediate with a low Gibbs free energy barrier of 0.33 eV. During the orbital overlap between the catalyst surface and the 2p orbital of the *COOH intermediate, p–d orbital hybridization in the Cu@ZnO/MXene composites promoted the formation of both σ and π bonds, thereby strengthening *COOH adsorption and accelerating the CO_2_ reduction reaction kinetics. As a result, the catalyst achieved an outstanding Faradaic efficiency of nearly 98.43% together with excellent operational stability over 20 h of continuous electrolysis [59].

## 5. Summary and Outlook

Despite the promising potential of Ti_3_C_2_T_x_ MXene-based composites for photocatalytic CO_2_ conversion, their practical development remains constrained by several challenges that extend beyond initial catalytic activity. In particular, photoinduced deactivation, dynamic changes in MXene–semiconductor interfaces, evolution of surface terminations, and the limited validation of performance under realistic reaction conditions require further investigation. Addressing these issues is essential for establishing reliable structure–activity relationships and developing durable photocatalysts for solar-driven CO_2_ conversion.

The stability of Ti_3_C_2_T_x_ under photocatalytic conditions is a critical concern because exposure to light, oxygen, and water can alter its structure and chemical composition. Importantly, degradation under illumination should be distinguished from oxidation during dark storage. Light-induced heating and photochemical processes may accelerate structural degradation, modify the electronic properties of MXene, and change its contribution to the overall photocatalytic reaction. Consequently, a composite that exhibits high initial activity may undergo progressive deactivation during prolonged irradiation.

A further complication is that the degradation of Ti_3_C_2_T_x_ may release carbon-containing species that overlap with the expected products of CO_2_ reduction. Therefore, product formation alone does not necessarily establish that the detected carbon originates from CO_2_. Future studies should systematically compare dark and illuminated conditions, with and without CO_2_, and include catalyst-free and semiconductor-only controls where appropriate. Isotopic labeling with ^13^CO_2_, combined with product analysis and post-reaction characterization, would provide stronger evidence for the origin of carbon-containing products. In parallel, protective surface layers, optimized MXene loading, and controlled interfacial architectures should be investigated to suppress uncontrolled degradation without compromising charge transfer or access to catalytic sites. The key objective is not simply to improve storage stability but to preserve catalytic function under sustained illumination and reaction conditions.

The interface between Ti_3_C_2_T_x_ and a semiconductor is often designed to promote charge separation and transfer through appropriate energy-level alignment and intimate interfacial contact. However, these interfaces may not remain unchanged during photocatalytic CO_2_ reduction. Prolonged irradiation, contact with water, local temperature changes, and reaction intermediates may induce interfacial oxidation, changes in bonding, structural rearrangement, or partial loss of contact between the components. Such evolution can alter the direction and efficiency of charge transfer and may cause the active interface to differ from the structure characterized before the reaction.

Future research should therefore move beyond static descriptions of heterojunctions and establish how their structures and electronic properties evolve during operation. Operando or in-situ spectroscopy, complemented by pre- and post-reaction microscopy, surface analysis, and electrochemical or photophysical measurements, can help identify changes in oxidation states, interfacial bonding, and charge-carrier behavior. Particular attention should be paid to distinguishing beneficial, controlled interfacial transformations from irreversible degradation. These studies are necessary to determine whether proposed Schottky junctions or other charge-transfer models remain valid under actual CO_2_ photoreduction conditions and to guide the design of interfaces that retain both activity and structural integrity over repeated cycles.

The surface terminations of Ti_3_C_2_T_x_, including –O, –OH, and –F, influence its electronic structure, surface polarity, adsorption behavior, and interactions with semiconductor components. Nevertheless, the termination composition established after synthesis or pretreatment may not represent the surface state present during photocatalysis. Contact with water, dissolved CO_2_, reaction intermediates, and prolonged illumination may cause changes in termination coverage or distribution and may expose or modify surface sites. These changes could influence CO_2_ adsorption and activation, proton-coupled electron transfer, intermediate binding, and product selectivity.

A major challenge is to establish a direct relationship between termination evolution and catalytic performance rather than assuming that the initial surface composition remains constant throughout the reaction. Future investigations should combine controlled termination engineering with in situ or operando characterization, supported by theoretical calculations that consider realistic surface compositions and reaction environments. The relative effects of –O, –OH, and –F should be evaluated alongside defects, interfacial bonding, and local reaction conditions. Such an approach may identify surface configurations that balance chemical stability, CO_2_ activation, and efficient charge transfer. Importantly, no single termination composition should be presumed universally optimal across different MXene compositions, semiconductor partners, and reaction systems.

Many laboratory studies employ controlled CO_2_ supplies, optimized catalyst loadings, and relatively short reaction periods. However, these conditions may not reflect practical applications, where CO_2_ partial pressure or concentration, water-vapor content, mass transfer, light intensity, and temperature can vary substantially. In gas–solid or gas–liquid–solid systems, limited CO_2_ delivery and competition between CO_2_ and water for surface sites may affect reaction rates and product selectivity. In addition, apparent activity measured over a short period may not reveal gradual deactivation, changes in selectivity, or the accumulation of degradation products.

Future studies should therefore evaluate MXene-based photocatalysts under well-defined and progressively more realistic conditions, including lower CO_2_ concentrations where relevant, controlled humidity or water supply, extended irradiation, and repeated-cycle or continuous-operation tests. Performance should be reported using clearly defined metrics, including product formation rates, selectivity, carbon balance, and stability over time, with the light source, intensity, reactor configuration, catalyst loading, and feed composition specified. Appropriate controls are particularly important for distinguishing genuine CO_2_ conversion from background carbon sources and catalyst decomposition. Scale-up studies should additionally assess batch-to-batch reproducibility, material recovery, and the durability of the catalyst architecture.

**Figure 7:**
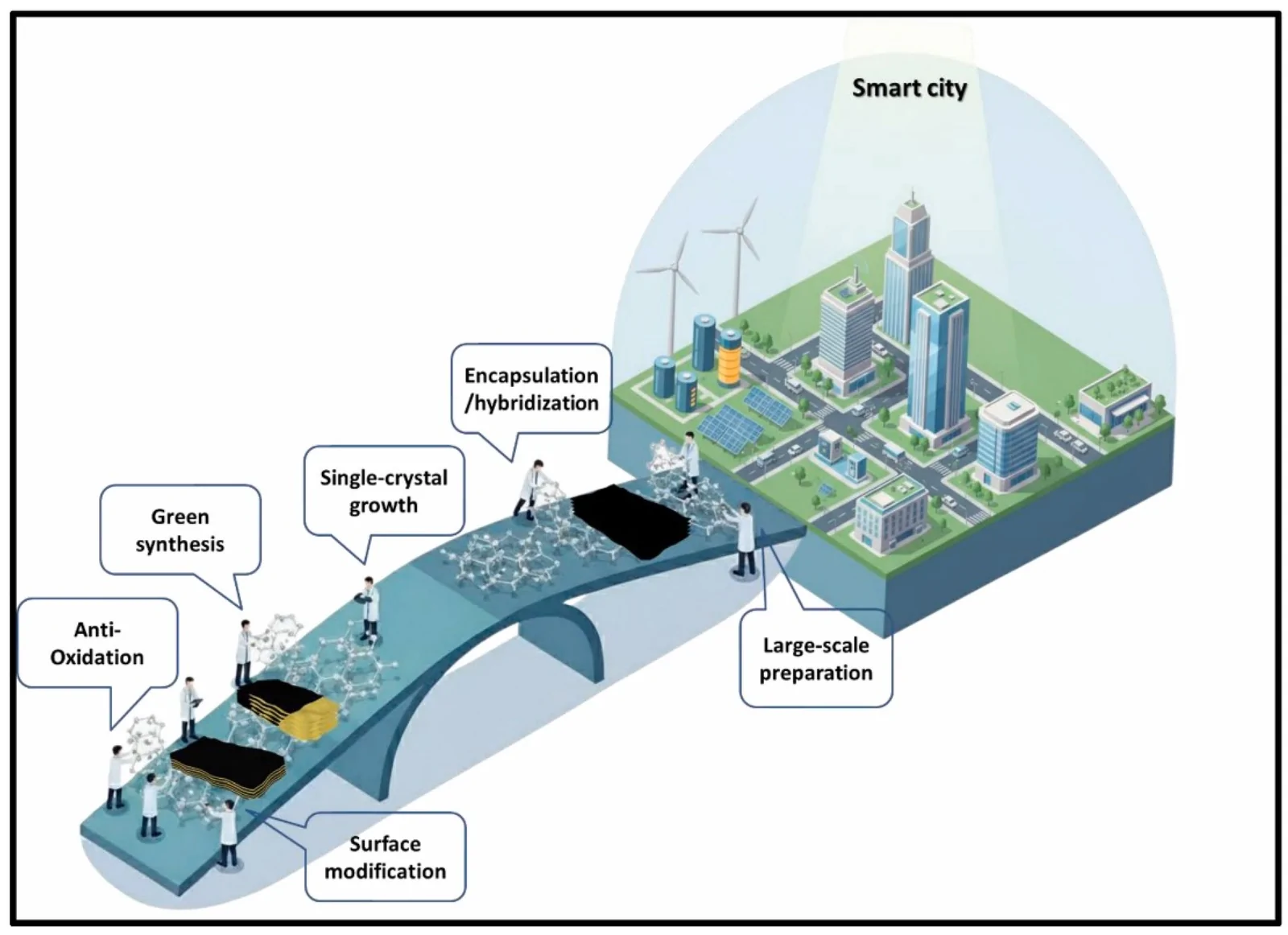
Roadmap for the development of two-dimensional and porous materials for energy storage, conversion, and environmental applications toward 2030. Reproduced from Ref. [60] under the terms of the Creative Commons Attribution (CC BY) licence.

As shown in Figure 7, future advances in Ti_3_C_2_T_x_-based photocatalysts will depend on integrating surface and interface engineering with rigorous stability assessment under reaction conditions. The rational construction of 0D/2D, 2D/2D, and 3D/2D heterostructures remains promising, provided that their charge-transfer mechanisms and structural evolution are experimentally validated. Ultimately, the most relevant design criterion is not the highest initial CO_2_ conversion rate alone, but the ability to sustain selective, verifiable CO_2_ reduction while preserving the active material and its interfaces during prolonged operation.

## 6. Conclusions

Ti_3_C_2_T_x_ MXenes are among the most extensively studied members of the two-dimensional MXene family and have attracted considerable scientific interest owing to their distinctive physicochemical, electrical, and optical properties. Although extensive research efforts have been devoted to MXenes in recent years, a comprehensive and systematic understanding of these materials has not yet been fully established. This review provides a fundamental overview of MXenes, covering their synthesis strategies, physicochemical properties, and emerging applications. We begin by surveying the preparation methods of MXenes, including both fluorine-containing and fluorine-free synthesis approaches. Subsequently, the key physicochemical characteristics of MXenes are systematically discussed to clarify the relationships between structure and performance. Finally, we summarize the diverse applications of MXenes in fields such as photocatalytic CO_2_ reduction and photoelectrochemical technologies. By analytically addressing these topics, this review aims to provide foundational knowledge for researchers entering the MXene field and to support the rational design and engineering of functional MXene-based composites for a broad range of advanced applications.

## Figures and Tables

**Table 1:** Comparison of MXene synthesis methods in terms of their advantages, limitations, morphological characteristics, and potential for large-scale production.

Synthesis Method	Strengths	Limitations	Effect on Morphology	Large-Scale Potential	Implications for Photocatalytic CO_2_ Reduction
Electrochemical etching	Green and relatively safe synthesis; low energy consumption	Low yield; morphology depends on electrolyte and applied potential	Produces etched multilayer MXene flakes. Electrochemical intercalation can facilitate delamination, although excessive etching may introduce defects and reduce flake size.	Marginally suitable	Electrolyte-dependent surface terminations and defects can modify surface polarity, CO_2_ adsorption, and interfacial charge transfer. Excessive defect formation or unfavorable termination coverage may impair charge transport or block active sites.
Alkaline etching	Efficient etching; produces hydrophilic MXenes with F-free surface terminations	Requires concentrated alkali and, in some protocols, high temperatures	Typically produces multilayer flakes with an expanded or accordion-like structure. The degree of delamination and layer separation depends on the etching conditions.	Marginally suitable	Oxygen-containing surface groups can modify surface polarity, electronic structure, and the adsorption of CO_2_-derived intermediates. Their catalytic effects depend on termination coverage, defect structure, and interactions with the coupled semiconductor.
Mechanical and electromagnetic-wave-assisted etching	Avoids strong acids; potentially rapid processing	Requires careful control of processing conditions; possible structural damage	Mechanical forces and electromagnetic-wave treatment can promote layer separation and exfoliation. However, excessive energy input may cause fragmentation, smaller lateral flake sizes, and structural defects.	Moderately suitable	Increased surface accessibility and defect formation may improve CO_2_ adsorption and expose reactive sites. However, excessive fragmentation or defects may impair charge transport. The effects of surface terminations must be assessed for each protocol.
Mechanical delamination	Simple process; avoids chemical etching	Low delamination yield; limited scalability	Produces few-layer or thin MXene flakes through mechanical separation. Prolonged or intensive treatment may reduce lateral flake size and introduce defects.	Unsuitable	Increased exposed surface area may improve access to CO_2_ and interfacial contact with semiconductors. However, delamination alone does not necessarily optimize adsorption energetics or charge transfer; these properties also depend on the inherited surface terminations.
HF etching	Simple and efficient etching process	Highly concentrated HF (approximately 50 wt% in some protocols); long etching time; severe safety and environmental concerns	Selective Al removal typically produces multilayer MXene with an accordion-like morphology. Subsequent intercalation and delamination can yield thin, few-layer or single-layer flakes.	Highly suitable (up to 50 g per batch)	Mixed terminations modify surface polarity, work function, and interfacial electronic structure. Oxygen-containing groups may favor particular adsorption configurations, while –OH groups can participate in hydrogen-bonding and proton-coupled processes. Fluorine groups also alter the local electronic environment. The overall effect on CO_2_ reduction depends on termination coverage, intermediate-binding energetics, and interfacial charge transfer.
Molten-salt etching	Effective for nitride MXenes and MAX precursors without Al; enables control of surface terminations	Delamination of multilayer MXene into single-layer or few-layer flakes remains challenging	Generally produces multilayer flakes with relatively compact or stacked structures. The morphology and surface features depend strongly on the salt composition, etching temperature, and precursor.	Moderately suit	Alternative termination chemistries may provide opportunities to tune work function, surface polarity, and interfacial charge redistribution. These changes can influence CO_2_ adsorption and intermediate stabilization. However, catalytic benefits must be established from the actual termination composition and photocatalytic measurements rather than assumed from the etching method alone.

**Table 2:** Reported band gap engineering of MXenes through surface functionalization with various terminal groups (–OH, –F, and –O).

MXene	Energy Band Gap Value
Sc_2_COH	0.45 eV
Mo_2_COH	0.10 eV
Cr_2_TiC_2_OH	0.84 eV
Sc_2_CF	1.03 eV
Mo_2_CF	0.25 eV
Cr_2_TiC_2_F	1.35 eV
Sc_2_CO	0.80 eV
Ti_2_CO	0.24 eV
Zr_2_CO	0.66 eV

**Table 3:** Comparison of MXene quantum dots and MXene nanosheets in photocatalytic systems.

Feature	MXene Quantum Dots (MQDs)	MXene Nanosheets (NSs)
Dimensionality & Size	Zero-dimensional, typically ultrasmall nanoparticles or quantum dots, generally <10 nm.	Two-dimensional, few-layer or multilayer nanosheets with extended lateral dimensions.
Active Sites & Edge Exposure	High edge-to-surface ratio, providing abundant exposed edge and defect-associated sites.	Large planar surfaces and relatively lower edge-to-area ratios; surface defects and terminations can be engineered to increase active sites.
Optical Properties	Size-dependent electronic and optical properties may arise from quantum confinement, depending on composition and size.	Broad optical absorption and high electrical conductivity; plasmonic responses, including LSPR-like behavior, have been reported for certain MXenes under specific conditions.
Charge Separation & Transport	Short charge-transport pathways and abundant surface/edge sites can facilitate interfacial electron transfer.	High in-plane electrical conductivity and extended conductive pathways facilitate electron transport; restacking may limit accessible surface area and interfacial charge transfer.
CO_2_ Reduction Role	Can provide abundant surface/edge sites and short diffusion pathways, potentially facilitating CO_2_ adsorption and interfacial reduction reactions.	Can function as an electron-transfer mediator, conductive support, or cocatalyst to promote charge separation and interfacial electron transfer.
Strategies to mitigate structural limitations	Anchor MQDs onto semiconductor surfaces, carbon supports, or porous frameworks; optimize surface functionalization and loading; and use controlled in-situ growth or self-assembly to improve dispersion.	Introduce porous spacers, carbonaceous materials, semiconductor nanoparticles, or molecular intercalants; construct 3D porous architectures; and use controlled exfoliation or in situ growth to preserve accessible interlayer spaces.
Preferred roles in photocatalytic CO_2_ reduction	Dispersed cocatalysts or sensitizing components that provide localized reaction sites, modify interfacial charge transfer, and potentially broaden the usable optical response.	Conductive cocatalysts, electron-transport mediators, supports for dispersed active sites, and components of 2D/2D heterojunctions that promote interfacial charge transfer and CO_2_ accessibility.
Potential advantages and limitations	Advantages: localized interfacial engineering, short transport distances, and potentially abundant accessible edge sites. Limitations: aggregation, difficult size control, and possible oxidation or surface-chemistry changes.	Advantages: extended conductive pathways, large interfacial contact areas, and versatile heterostructure construction. Limitations: restacking, reduced accessibility of interlayer surfaces, and potentially longer transport paths across poorly connected interfaces.

**Table 4:** Representative studies of MXene-based photocatalysts for CO_2_ reduction toward CO, CH_4_, CH_3_OH, and HCOOH: catalyst composition, light irradiation, reaction conditions, product yield.

MXene-Based Composites	Reaction System	Main Products	Yield (μmol·g^−1^·h^−1^)	Selectivity (%)	Light Source	Remarks on Performance Enhancement	Ref.
CuInS_2_/Ti_3_C_2_T_x_	TEOA solution bubbled with CO_2_	CH_3_COOH	250.0	97.5	Xenon light (300 W)	MXene–semiconductor interfacial coupling may promote charge separation and electron transfer; product identity and selectivity mechanism should be verified against the original study.	[39]
Bi_2_O_2_SiO_3_/Ti_3_C_2_T_x_	H_2_O bubbled with CO_2_	CO	17.82	74.2	Xenon light (300 W)	Composite-interface engineering may facilitate charge transfer, while the oxide–silica framework may modify surface accessibility and CO_2_ adsorption.	[30]
COF-367/Ti_3_C_2_T_x_	β-NAD+ and TEOA and a phosphate-buffered solution (100 mM, pH = 8)	HCOOH	1880.0	100.0	simulated sunlight (AM 1.5100 mW cm^−2^	2D/2D COF–MXene heterointerfaces promote charge separation and transfer; the coupled photoenzymatic system supports selective formate production.	[40]
La_2_Ti_2_O_7_/Ti_3_C_2_T_x_	CO_2_ and H_2_O	CH_4_	11.16	66	Xenon light (300 W)	MXene–oxide interfacial coupling may improve charge transport and surface reaction kinetics. Verify the specific role of the La_2_Ti_2_O_7_ phase.	[41]
C_3_N_5_/Ti_3_C_2_T_x_	β-NAD+ and TEOA and a phosphate buffer (100 mM, pH = 7.5)	HCOOH	3510.0	100.0	simulated sunlight (AM 1.5100 mW cm^−2^	Interfacial charge transfer in the coupled carbon nitride–MXene system may improve electron utilization and formate selectivity.	[42]
CeO_2_/Ti_3_C_2_T_x_	H_2_O bubbled with CO_2_	C_2_H_5_OH	6127	98	Solar simulator (150 W)	Ce^3+^/Ce^4+^ redox chemistry, CO_2_ adsorption, and MXene-assisted charge transfer act synergistically; verify the yield basis and reaction duration.	[43]
TiO_2_/Ti_3_C_2_T_x_	TEOA, NAD^+^, and [Cp*Rh]	HCOOH	767.0	77.4	Xenon light (300 W)	MXene–TiO_2_ interfacial charge transfer combined with the NAD^+^/[Cp*Rh]-associated cofactor-regeneration system supports formate production.	[44]
Bt/C_3_N_4_/Ti_3_C_2_T_x_	H_2_O bubbled with CO_2_	CH_4_	955.0	72.4	HID lamp (35 W)	Multicomponent heterostructure engineering may enhance charge separation and light utilization. Verify the specific role of Bt and the proposed reaction pathway.	[45]
Co-Co LDH/Ti_3_C_2_T_x_	TEOA solution bubbled with CO_2_	CO	1250.0	63.9	LED lamp (5 W)	Co-containing catalytic sites and MXene-assisted electron transport may synergistically promote CO_2_ reduction to CO.	[46]
g-C_3_N_4_/Ti_3_C_2_T_x_	Methanol, CO_2_ and H_2_O	CO	306.8	80.2	HID Car lamp (35 W)	MXene–carbon nitride interfacial coupling can facilitate charge separation and electron transport to CO_2_-reduction sites.	[47]
SA Co/Ti_3_C_2_T_x_	CH_3_CN, H_2_O, TEOA, CO_2_	CO	6060.0	72.9	Xenon light (300 W)	Isolated Co sites provide defined catalytic centers, while MXene supports electron transport and may influence the adsorption of CO_2_-derived intermediates.	[48]
CeO_2_/TiO_2_/Ti_3_C_2_T_x_	CO_2_ and H_2_O	CH_4_	0.22	70.7	Xenon light (300 W)	Multicomponent heterostructure formation may facilitate charge separation and interfacial transport. Confirm the reported yield unit and the specific contribution of each component.	[49]
Cs_2_AgBiBr_6_/Ti_3_C_2_T_x_	CO_2_ and H_2_O	CO	11.1	70.9	Xenon light (150 mW cm^−2^)	MXene–double-perovskite interfacial coupling may promote charge extraction and transfer to CO_2_-reduction sites.	[50]

Note: The reported photocatalytic yields and production rates were obtained under different experimental conditions, including variations in light source, irradiation intensity, catalyst loading, reaction duration, sacrificial agent, solvent system, and CO_2_ delivery conditions. Therefore, the reported values should not be directly compared across studies or used to establish an absolute ranking of catalytic activity. Cumulative product yields (e.g., μmol g^−1^) should be distinguished from time-normalized production rates (e.g., μmol g^−1^h^−1^). All values are reproduced from the respective original references, and comparisons should be made only when the relevant experimental conditions and normalization bases are sufficiently comparable.

## Data Availability

No new data were created or analyzed in this review study. Data sharing does not apply to this article.
